# Associations between Methylation of *Paternally Expressed Gene 3* (*PEG3*), Cervical Intraepithelial Neoplasia and Invasive Cervical Cancer

**DOI:** 10.1371/journal.pone.0056325

**Published:** 2013-02-13

**Authors:** Monica D. Nye, Cathrine Hoyo, Zhiqing Huang, Adriana C. Vidal, Frances Wang, Francine Overcash, Jennifer S. Smith, Brandi Vasquez, Brenda Hernandez, Britta Swai, Olola Oneko, Pendo Mlay, Joseph Obure, Marilie D. Gammon, John A. Bartlett, Susan K. Murphy

**Affiliations:** 1 Division of Gynecologic Oncology, Department of Obstetrics and Gynecology, Duke University School of Medicine, Durham, North Carolina, United States of America; 2 Department of Obstetrics and Gynecology, Program of Cancer Detection, Prevention and Control, Duke University School of Medicine, Durham, North Carolina, United States of America; 3 Duke Comprehensive Cancer Center, Duke University Medical Center, Durham, North Carolina, United States of America; 4 Department of Epidemiology, University of North Carolina at Chapel Hill, Chapel Hill, North Carolina, United States of America; 5 Kilmanjaro Christian Medical Center, Moshi, Tanzania; 6 Cancer Research Center of Hawaii, University of Hawaii, Honolulu, Hawaii, United States of America; 7 Department of Pathology, Kilimanjaro Christian Medical Centre, Tumaini University, Moshi, Tanzania; 8 Department of Obstetrics and Gynecology, Kilimanjaro Christian Medical Centre, Tumaini University, Moshi, Tanzania; 9 Division of Infectious Diseases, Department of Medicine and Duke Global Health Institute, Duke University School of Medicine, Durham, North Carolina, United States of America; 10 Lineberger Comprehensive Cancer Center, University of North Carolina at Chapel Hill, North Carolina, United States of America; 11 Duke Women's Health Collaboration, Durham, North Carolina, United States of America; Università di Napoli Federico II, Italy

## Abstract

Cytology-based screening for invasive cervical cancer (ICC) lacks sensitivity and specificity to discriminate between cervical intraepithelial neoplasia (CIN) likely to persist or progress from cases likely to resolve. Genome-wide approaches have been used to identify DNA methylation marks associated with CIN persistence or progression. However, associations between DNA methylation marks and CIN or ICC remain weak and inconsistent. Between 2008–2009, we conducted a hospital-based, case-control study among 213 Tanzania women with CIN 1/2/3 or ICC. We collected questionnaire data, biopsies, peripheral blood, cervical scrapes, Human papillomavirus (HPV) and HIV-1 infection status. We assessed *PEG3* methylation status by bisulfite pyrosequencing. Multinomial logistic regression was used to estimate odds ratios (OR) and confidence intervals (CI 95%) for associations between *PEG3* methylation status and CIN or ICC. After adjusting for age, gravidity, hormonal contraceptive use and HPV infection, a 5% increase in *PEG3* DNA methylation was associated with increased risk for ICC (OR = 1.6; 95% CI 1.2–2.1). HPV infection was associated with a higher risk of CIN1-3 (OR = 15.7; 95% CI 5.7–48.6) and ICC (OR = 29.5, 95% CI 6.3–38.4). Infection with high risk HPV was correlated with mean *PEG3* differentially methylated regions (DMRs) methylation (r = 0.34 p<0.0001), while the correlation with low risk HPV infection was weaker (r = 0.16 p = 0.047). Although small sample size limits inference, these data support that *PEG3* methylation status has potential as a molecular target for inclusion in CIN screening to improve prediction of progression.

**Impact statement:**

We present the first evidence that aberrant methylation of the *PEG3* DMR is an important co-factor in the development of Invasive cervical carcinoma (ICC), especially among women infected with high risk HPV. Our results show that a five percent increase in DNA methylation of *PEG3* is associated with a 1.6-fold increase ICC risk. Suggesting *PEG3* methylation status may be useful as a molecular marker for CIN screening to improve prediction of cases likely to progress.

## Introduction

Approximately half a million women throughout the world are diagnosed with cervical cancer annually and slightly over half of these women die from the disease; 80% are diagnosed in resource-poor settings [1]. Cytology-based screening and aggressive treatment of pre-cancerous lesions are the most widely used strategies for preventing invasive cervical cancer (ICC) worldwide. Human papillomavirus (HPV), the only known etiologic agent for ICC, has been used to further stratify CIN cases from women with normal cytology, with high sensitivity [2] but low specificity. Overall, approximately 4–10% of women with normal cytology are HPV DNA positive, and thus the sensitivity and specificity for HPV DNA testing remains suboptimal, resulting in a non-negligible number of women with false positive results, requiring follow-up at cost to both the health care system and the patient. Suboptimal sensitivity and specificity also has been shown to decrease adherence to recommended follow-up visits [3]. The use of cofactors previously associated with CIN or ICC such as age, parity, cigarette smoking, Chlamydia trachomatis infection, and long-term hormonal contraceptive use has not yielded additional insights for discriminating among CIN1 cases likely to persist or progress from those likely to regress. Thus, identifying specific molecular features that can improve prediction of which CIN cases are likely to progress to ICC remains a priority.

Epigenetic mechanisms of gene regulation, including DNA methylation, have an important role in coordinating gene expression changes in response to viral infection [4]. Epigenetic changes are also proposed as a driving force in the carcinogenic process that may be involved in the trajectory of HPV infections progressing from CIN to ICC [5] [6], [7]. However, the identity of such a specific epigenetic target(s) is still unknown. *PEG3* is a paternally expressed imprinted gene on chromosome 19q13.43 that encodes a protein with tumor suppressive function that plays a role in facilitating p53/c-*myc*-mediated apoptosis. *PEG3* is regulated by allele-specific DNA methylation whereby only the maternally derived allele is normally methylated. Hypermethylation at the *PEG3* regulatory differentially methylated region (DMR) leads to a decrease in *PEG3* transcription, which in turn is presumed to inhibit the pro-apoptotic function of this gene [8], [9]. Aberrant methylation at this DMR has been associated with lower levels of *PEG3* expression of this tumor suppressor as has been observed in other gynecologic cancers, including such as ovarian and endometrial cancers [10] [11] [12]. In addition, *PEG3* DMR hypermethylation and transcriptional silencing has also been shown to occur in gliomas [13] [14]. These results together suggest that the PEG3 zinc finger protein may function as a tumor suppressor gene in cancer, and may be particularly relevant to cancers affecting the female reproductive tract. We therefore sought to determine if and how methylation changes at the regulatory *PEG3* DMR are associated with HPV infection, CIN and ICC.

## Methods

### Study Participants

Between November 2008 and March 2009, eligible study participants were recruited from the Reproductive Health Clinic (RHC) at KCMC, a Cervical Cancer prevention clinic funded by the World Health Organization. Methods for participant identification and enrollment have been previously described [15]. Briefly, inclusion criteria were women aged 18 years or older with no prior history of an abnormal Pap test. Some of the participants were patients with suspicious ICC lesions referred to KCMC for an open and colposcopic directed biopsy. Of the 250 women enrolled, two refused to participate resulting in a 99% response rate. Of the remaining 248 there were 14 where we were unable to determine cancer diagnoses and 21 without HPV results. The final study population comprised 213 women with questionnaire, CIN/ICC, HIV-1 status, and HPV genotype data. Thus, cases were women with any grade of CIN1/2/3 or ICC, and controls were women without CIN or ICC as assessed during a cytology-based screening visit.

### Ethics Statement

Written informed consent was obtained from each study participant prior to enrollment. Research Ethics Boards at Kilimanjaro Christian Medical Centre (KCMC), the University North Carolina at Chapel Hill and Duke University approved this study.

### Data Collection

#### Questionnaires

A trained nurse-interviewer obtained informed consent and administered a standardized in-person 40-minute questionnaire. Socio-demographic characteristics collected included age, martial status, type of marriage (polygamy vs. monogamy), tribe, educational attainment, cigarette smoking, alcohol consumption, reproductive history (e.g. menarche, parity and gravidity), sexual history (e.g. lifetime number of sexual partners, age at first intercourse), and medication and supplement use.

#### Specimens

Two cervical scrapes were obtained from each participant. One was prepared on a glass slide for cytological evaluation for CIN and ICC diagnosis, data also used as outcome for this study. The second specimen was collected using a cytobrush and rinsed into Preserv-Cyt ™ media (Hologic, Inc Malborough, MA). One-third of the specimen was reserved for HPV analysis and stored at 4°C, and the remaining two-thirds were centrifuged to pellet the cells, which were stored as aliquots at −80°C. DNA extracted from these cells was later used for DNA methylation analysis. Biopsy specimens were collected only when clinically indicated. Routine cervical screening by visual inspection with acetic acid (VIA) was performed. If there were positive findings by VIA or through direct examination, the patient was triaged and treated accordingly. Patients with negative findings were given follow up appointments within two weeks to provide results.

#### Ascertainment of the study outcomes: CIN and carcinoma

The pathologist at KCMC (BS) processed and read the Papanicolaou smears and biopsy specimens using standard conventions according to ASCCP guidelines as appropriate (http://www.asccp.org/). A gynecologist (BV) also reviewed medical charts monthly for HIV-1 test and cyto-pathological results to classify the cases using the Bethesda classification system. The results were then coded based on pathology and medical record findings. They were coded as 1) no evidence of cytological abnormality, 2) mild dysplasia including LSIL and CIN1, 3) moderate dysplasia including HSIL and CIN2-3, or 4) cancer that was primarily squamous cell carcinoma with the exception of three adeno-squamous carcinomas of the uterine cervix. None of the specimens were classified as atypical cells of uncertain significance (ASCUS). The results were available via the patient's clinic records, and the pathologist entered them into the database. The records were then compiled and securely shared with Duke University.

#### HPV genotyping

ThinPrep® specimens and homogenized biopsy specimens were shipped to the University of Hawaii Cancer Center. DNA was extracted and amplified by PCR targeting of a 450 bp region within the HPV L1 gene using the PGMY09/PGMY11 primers [16]. The human β-globin gene was used as an internal control for sample accuracy. We were able to obtain viral DNA analysis for all patient specimens. HPV-positive specimens were genotyped using the HPV Linear Array® (Roche Molecular Systems Inc., Branchburg, NJ, USA).

#### Ascertainment of HIV-1 infection status

Plasma and buffy coat were isolated via centrifugation of peripheral blood specimens collected in EDTA-containing vacutainer tubes from the patients. Two rapid HIV-1 tests were used to analyze the plasma samples for HIV-1 status (Capillus HIV-1/HIV-2, Trinity Biotech PLC, Bray, Country Wicklow, Ireland and Determine HIV-1/2, Abbott Laboratories, Abbott Park, IL). The standard clinical practice of Western blot was used for specimens that were reactive (Genetic Systems HIV-1 Western blot kit; Bio-Rad, Hercules, CA).

#### PEG3 methylation analysis

Genomic DNA was prepared from cells isolated from the cervical scrapes or from biopsy specimens using PureGene protocol reagents (Qiagen; Valencia, CA) and treated with sodium bisulfite using the Zymo Easy-96 DNA methylation kit (Zymo Research, Irvine, CA). Bisulfite treatment modifies the DNA by converting unmethylated cytosines to uracils, and leaves methylated cytosines unchanged. Pyrosequencing was performed using a Qiagen Pyromark Q96 MD Pyrosequencer.

The DMR analyzed is located within the *PEG3* promoter region at chromosome 19q13.43. The pyrosequencing assay for *PEG3* was used as previously described [12] with the exception that a 63°C annealing temperature was used for the PCR reaction. Genomic coordinates for the region amplified by PCR are chr19: 57,351,945–57,352,096 (UCSC Genome Browser, GRCh37/hg19). The performance of the assay was assessed using defined mixtures of unmethylated and methylated bisulfite modified genomic DNA (i.e., 0%, 25%, 50%, 75% 100% methylated; Epitect Bisulfite Controls; Qiagen).

### Statistical Analyses

The mean DNA methylation fractions at the individual CGs were analyzed and compared among controls, women without CIN1/2/3 or ICC (n = 147) and the three case groups (CIN1 [n = 21], CIN2/3 [n = 17], and ICC [n = 48]) using F-tests. Principal components analyses (PCA) were applied to determine if a single mean represents the methylation fraction at the CG dinucleotides within the DMR region. Methylation of the CGs was sufficiently correlated thus allowing a single mean to be used. F-tests were used to determine if DNA methylation at individual CGs within *PEG3* DMR differed significantly by infection with high risk (HR) versus low risk (LR) HPV and other HPV genotypes. Classification of high risk and low risk genotypes was based on FDA-approved HPV molecular tests (CDC). HR HPV genotypes included HPV 16,18, 31, 33, 35, 39, 45, 51, 52, 56, 58, 59,59 and LR HPV included HPV 6, 11, 26, 66, 68, 70, 73 and, 82 as described previously [15]. Women who tested positive for at least one HPV genotype based on oncogenic risk were categorized as HR-HPV, LR-HPV or other HPV.

To estimate odds ratios (OR) and corresponding 95% confidence intervals (CIs) for the association between CIN/ICC and changes in DMR methylation, we used multi-polynomial logistic regression models. All models included methylation using 5% increments with adjustments made for HPV-infection, pregnancy history, HIV-1 status, and hormonal contraceptive use. Aberrant methylation was defined as <25^th^ or >75^th^ quartiles, since both hypermethylation and hypomethylation of this locus can lead to deregulation of *PEG3* expression and lead to loss of imprinting [11] [12] [17]. We used chi-square analysis to test for potential confounders with CIN and cancer status. All statistical analyses were conducted in SAS 9.1 (SAS Institute, Cary, NC).

## Results

### Study Population Characteristics

Case groups and controls differed significantly by age, HPV infection and gravidity (Table 1). The mean age among controls was 40.3 years (SD = 9.9). Among case groups the average age increased with severity of lesion (35.7 years, SD = 12.2 for CIN1; 44.7 years, SD = 9.8 for CIN2/3 and 55.2 years, SD = 12.3 for ICC; p<0.001). The prevalence of HPV infection also increased with increasing severity of lesion, as 67% of CIN1, 88% CIN2/3 and 89% of ICC had detectable HPV infection (p-value<0.0001). Gravidity was high, with 94% of controls and 86% of women with CIN1 reporting that they had ever been pregnant as compared to 100% of the women with CIN2/3 or ICC. Long term OC use was similar in CIN1 and controls 76% and 68%, respectively, but was significantly lower among higher grades of CIN (59%) and ICC (40%).

**Table 1:** Distribution of participant characteristics by case-control status.

Characteristics	Control	CIN1	CIN2/3	ICC*
	N = 147	N = 21	N = 17	N = 48
**Mean Age (s.d.)**	40.3 (9.9)	35.7 (12.2)	44.7 (9.8)	55.2(12.3)
**Ever Pregnant**	**N (%)**	**N (%)**	**N (%)**	**N (%)**
Yes	135 (94.8)	18 (85.7)	17 (100)	48 (100)
No	12 (9.2)	3 (14.3)	0 (0)	0 (0)
**Oral Contraceptives**	**N (%)**	**N (%)**	**N (%)**	**N (%)**
Yes	98 (66.6)	16 (76.2)	10 (58.8)	19 (39.6)
No	47 (31.9)	5 (23.8)	7 (41.2)	29 (60.4)
Missing	2 (1.5)	0 (0)	0 (0)	0 (0)
**Any HPV**	**N (%)**	**N (%)**	**N (%)**	**N (%)**
≥1 HPV	20 (13.6)	12 (57.1)	14 (82.4)	33 (68.8)
No HPV	122 (83)	6 (28.5)	2 (11.8)	4 (8.3)
Missing	5 (3.4)	3 (14.3)	1 (5.8)	11 (22.9)

HPV type Prevalence	Single/Multiple	Single/Multiple	Single/Multiple	Single/Multiple
High Risk	4/23	4/18	3/19	8/68
Low Risk/Other	4/19	0/15	1/33	0/25

* ICC/Cancer subtype: 45 patients had squamous cell carcinoma; 3 had adenosquamous carcinomas.

High Risk HPV- 16, 18, 31, 33, 35, 39, 45, 51, 52, 56, 58, 59, 66, 68.

Low/Other Risk HPV – 6,11, 26, 40, 42, 55, 61, 62, 69, 70, 72, 73, 81, 82, 83, 84.

### *PEG3* pyrosequencing assay validation

**Figure 1:**
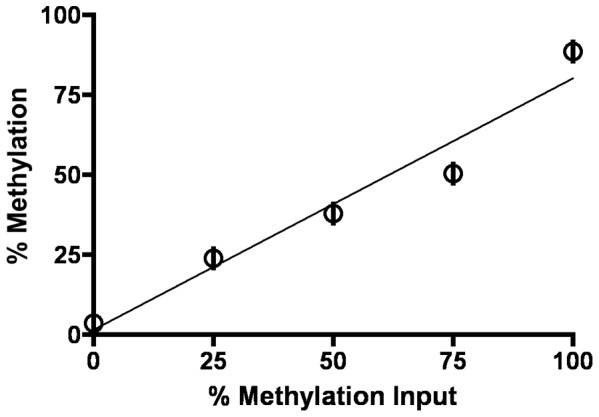
Validation of the *PEG3* pyrosequencing assay. Defined mixtures (x-axis) of methylated and unmethylated DNAs were prepared and analyzed in quintuplicate by pyrosequencing (y-axis). The results shown represent the mean; error bars indicate standard deviations. The Pearson rho is 0.953 with a p-value of 0.004.

We first validated the performance of the pyrosequencing assay in quintuplicate using fully methylated and unmethylated DNAs in defined proportions. At each increase in the amount of methylated DNA input, there was also an increase in the amount of methylation measured (Pearson rho = 0. 953; p = 0.004). The average standard deviation between these replicate measures was 1.59% (range, 0.34% to 3.47%). These results indicate that this assay has the ability to reproducibly detect differences in methylation values (Figure 1).

### Association between HPV infection status and DNA methylation at *PEG3* DMRs

We examined methylation fractions of 10 CpG sites within the *PEG3* DMR in relation to HPV genotype. As shown in Table 2, among women infected with at least one HR-HPV genotype, we found a moderate correlation between methylation of all 10 CpG sites within the *PEG3* DMR and HPV infection (r = 0.34, p-value <0.0001; range = 0.32 to 0.41). LR-HPV genotypes had a sizably weaker correlation with *PEG3* methylation (r = 0.061, p value = 0.552). Infection with other HPV genotypes was not correlated with methylation fractions within the *PEG3* DMR (mean correlation coefficient = 0.0163 p = 0.876).

**Table 2:** Correlation between High and Low Risk HPV status and methylation fraction means at differentially methylated region (DMRs) of *PEG3*.

Chromosomal region and CpG site	High Risk HPV	Low Risk HPV	Other HPV
*PEG3* (N = 149)	Correlation coefficient (p-value)	Correlation coefficient (p-value)	Correlation coefficient (p-value)
**CpG1**	0.40 (<0.0001)	−0.01 (0.90)	0.00 (0.98)
**CpG2**	0.35 (<0.0001)	0.02 (0.86)	0.00 (0.98)
**CpG3**	0.37 (<0.0001)	0.03 (0.74)	0.13 (0.23)
**CpG4**	0.32 (<0.0001)	−0.01 (0.94)	0.01 (0.93)
**CpG5**	0.41 (<0.0001)	−0.04 (0.67)	0.03 (0.77)
**CpG6**	0.33 (<0.0001)	−0.04 (0.68)	0.02 (0.85)
**CpG7**	0.36 (<0.0001)	−0.01 (0.89)	0.003 (0.98)
**CpG8**	0.33 (<0.0001)	0.001 (0.99)	0.05 (0.65)
**CpG9**	0.36 (<0.0001)	−0.04 (0.72)	0.02 (0.85)
**CpG10**	0.37 (<0.0001)	−0.01 (0.92)	−0.01 (0.94)
**Mean**	0.34 (<0.0001)	0.06 (0.55)	0.02 (0.87)

High Risk HPV - 16,18, 31, 33, 35, 39, 45, 51, 52, 56, 58, 59, 66, 68.

Low/Other Risk HPV – 6, 11, 26,40, 42, 55, 61, 62, 69, 70, 72, 73, 81, 82, 83, 84.

### *PEG3* DMR methylation, CIN and ICC

Table 3 summarizes the odds ratios (ORs) and 95% CI for the association between *PEG3* DMR methylation and CIN and ICC status, adjusted for age, hormonal contraceptive use and any HIV-1 infection. We found little or no association between mean *PEG3* methylation status and CIN1/2/3, (OR = 1.03; 95% CI (0.79–1.33); p-value = 0.80). However, a 5% increase in methylation levels at the *PEG3* DMR was associated with a nearly two-fold increase in the risk of ICC (OR = 1.6; 95% CI 1.2–2.1; p-value = 0.0003). As expected, infection with any HPV genotype was also associated with a higher risk of CIN1-3 (OR = 15.7 95% CI(5.1–48.6)) and ICC (OR = 29.5, 95% CI (6.3–38.4)).

**Table 3:** Adjusted odds ratios for the associations between mean DMR methylation for *PEG3*, HPV status and CIN and ICC.

	*PEG3* mean (s.d.)	*OR, (95% CI), p-value
**Control**	39.0 (2.8)	Ref
**CIN**	39.2 (5.0)	1.0 (0.79–1.33) p = 0.8
**ICC**	45.5 (5.8)	1.6 (1.2–2.1) p = 0.0003

	HPV (None/Any)	OR, (95% CI)
**Control**	122/20	Ref
**CIN**	8/26	15.7 (5.1–48.6)
**ICC**	4/33	29.5 (6.3–38.4)

* Controlling for HIV-1 status, HPV positive status, age, and oral contraceptive (OC) use.

Per 5% methylation increase.

## Discussion

Our key findings in this case-control study of Tanzanian women are that after adjusting for HPV infection, age, OC use, and HIV-1 status, a 5% increase in DNA methylation at the *PEG3* DMR was associated with a 1.6 fold increase in ICC risk. We also found that *PEG3* DMR hypermethylation was correlated with HPV infection; a correlation that was stronger for high risk as compared to low risk HPV infection. As would be expected, HPV infection was associated with increased risk of CIN1/2/3 and ICC. We present the first evidence in support of the hypothesis that aberrant methylation of the *PEG3* DMR is an important co-factor in the development of ICC, especially among women infected with HR HPV.

Our data suggests that increasing grade of lesion from CIN to ICC correlates with HPV infection and *PEG3* DMR hypermethylation and is consistent with DNA methylation-mediated repression of *PEG3* as found in previous studies. Hypermethylation of various genes (i.e. *MGMT*, *FHIT*, *GSTP1*, and *MHL1*) in ICC case control sudies has been reported [18] [19] [20]. Previous studies have reported DNA methylation changes and HPV status in head and neck squamous cell cancer [21]. These findings support the idea that the presence of aberrantly methylated genes could be used as a relatively sensitive and specific screening assay to detect CIN and ICC. These previous studies did not investigate *PEG3*. To our knowledge this is the first study done with a human population that investigated the relationship between *PEG3* DMR status and HPV infection and how this plays a role in CIN and ICC. Although cause and effect cannot be established in this case-control study, our findings suggest that *PEG3* DMR methylation is a potential mechanism by which susceptibility to progression to ICC may occur, and thus may be a useful marker to identify CIN cases likely to progress.

The mechanisms by which *PEG3* DNA methylation increases risk of ICC are unclear. However, there is evidence suggesting that *PEG3* plays an important biological role in p53/c-myc mediated apoptosis, implicating *PEG3* as a gene whose function may be in part to prevent carcinogenesis [8] [9]. The p53-mediated apoptosis pathway has two potential outcomes: induction of a) growth arrest or b) cell death; Peg3 has been shown to play a role downstream of p53 activating apoptosis via its interaction with Bax. Peg3 interacts with Bax, resulting in apoptosis [8]. These prior reports, together with our findings, support the hypothesis that *PEG3* functions as an important tumor suppressor in carcinogenesis.

The association found here is consistent with findings from *in vitro* and *in vivo* studies showing that the *PEG3* promoter is hypermethylated with consequent transcriptional repression in ovarian and endometrial cancers [12] [11]. In cervix, ovarian, and endometrial cancer cell lines *PEG3* is silenced suggesting that during carcinogenesis, hypermethylation may be selected for in order to inhibit the pro-apoptotic function of PEG3. Our case control study shows an association between hypermethylation of *PEG3* and ICC but not CIN, suggesting that these methylation alterations take place during transformation rather than in pre-cancerous lesions. Alternatively, the attenuation in risk may be due to combining low grade CIN largely comprised of lesions likely to regress, with higher-grade CIN cases, the majority of which have potential to progress and become ICC. Intriguingly, the correlation of HPV infection, an etiologic agent of ICC, and *PEG3* hypermethylation is consistent with a multi-step process that starts with epigenetic mechanisms and HPV infection.

The main limitation of this study is the small sample size to examine *PEG3* DMR methylation in relation to grade specific CIN, after accounting for the effect of HPV infection. It is possible that our inability to find associations between *PEG3* methylation and CIN was due to combining CIN (in whom the majority or women are likely to regress) and CIN2 and CIN3 (in whom a smaller proportion persist or progress) [22]. However, we had adequate statistical power to evaluate *PEG3* and ICC risk. Another limitation is the case-control design, limiting our ability to infer *PEG3* methylation as an important factor in progression. However, identifying methylation marks associated with case-control differences is a necessary step allowing for examination of this marker in longitudinal studies currently under way by several groups [23].

Despite these limitations, we found hypermethylation of the *PEG3* DMR increased the risk for ICC after adjusting for known confounders. We also found a strong correlation between HPV genotype and DNA methylation at the *PEG3* DMR. Cytosine methylation is a stable modification in human tissue samples, and therefore *PEG3* DMR methylation status could potentially be used as a marker to identify CIN likely to progress to ICC. Larger studies in a more diverse study population are required to replicate these findings.
